# Bioinformatics, expression analysis, and functional verification of allene oxide synthase gene *HvnAOS1* and *HvnAOS2* in qingke

**DOI:** 10.1515/biol-2022-0855

**Published:** 2024-04-25

**Authors:** Likun An, Ziao Wang, Yongmei Cui, Youhua Yao, Yixiong Bai, Yuehai Liu, Xin Li, Xiaohua Yao, Kunlun Wu

**Affiliations:** Academy of Agricultural and Forestry Sciences, Qinghai University, Xining, Qinghai 810016, China; Laboratory for Research and Utilization of Qinghai Tibet Plateau Germplasm Resources, Xining, Qinghai 810016, China; Qinghai Key Laboratory of qingke Genetics and Breeding, Xining, Qinghai 810016, China; Qinghai Subcenter of National qingke Improvement, Xining, Qinghai 810016, China

**Keywords:** qingke, allene oxide synthase, *HvnAOS1* and *HvnAOS2* genes, bioinformatics analysis, molecular docking simulation

## Abstract

Allene oxide synthase (AOS) is a key enzyme involved in the jasmonic acid (JA) synthesis pathway in plants. To explore its function on the regulatory mechanism of JA synthesis, we screened and identified two *AOS* genes *HvnAOS1* and *HvnAOS2* in qingke. Both HvnAOS1 and HvnAOS2 contained conserved heme-binding motif, which is most closely related to AtsAOS2, indicating controlled dehydration of fatty acid hydroperoxides to allene oxides. Molecular docking simulations identified the key amino acid sites that were important for heme binding and interaction with 13(*S*)-HPOT, respectively. The expression pattern also indicated that *HvnAOS1* and *HvnAOS2* were highly induced by JA, abscisic acid, and salicylic acid. Subcellular localization of *HvnAOS1* and *HvnAOS2* using transient expression of *Agrobacterium tumefaciens* showed the green fluorescent protein signal in the cell cytoplasm of the *N*. *benthamiana* leaves. Overexpression of *HvnAOS1* and *HvnAOS2* in *Arabidopsis aos* mutant restored male fertility and plant resistance to *Botrytis cinerea*, indicating that *HvnAOS1* and *HvnAOS2* can restore the functions of *AOS* in *Arabidopsis aos* mutant.

## 1 Introduction

Qingke (*Hordeum vulgare* L. var. *nudum* Hook. f.) is the most important grain and fodder crop, with the advantages of early maturity, easy cultivation, and tolerance to multiple adversities in the Qinghai-Tibetan Plateau of China [1–3]. Qingke seeds are rich in nutrients and can be processed into a variety of foods, and are also an important raw material for winemaking, which is popular among people in the Qinghai-Tibetan Plateau [4–6]. Due to the extremely harsh environment in the Qinghai-Tibetan Plateau region, such as drought, salinity, low temperature, and strong ultraviolet radiation, the production of qingke is subject to long-term comprehensive adversity stresses [4,7,8]. So, how to improve the resistance to integrated adversities in the plateau region is of great significance for qingke production in the Qinghai-Tibetan Plateau.

As a kind of growth regulator, jasmonic acid (JA) and its derivatives (JAs) play an important role in plant growth and development and various physiological activities [9–12]. In addition, JA can act as the most responsive signaling molecules to be in response to comprehensive stress, such as drought, salinity, high and low temperature, pests and diseases, mechanical damage, strong UV radiation, etc. [13–15]. Allene oxide synthase (AOS) is the key enzymes in the JA synthesis pathway, and its substrate is 13(*S*)-hydroperoxy-linolenic acid (13(*S*)-HPOT). The core region of AOS contains a heme molecule, and after 13(*S*)-HPOT enters the core region of AOS, it is converted to unstable 12(*S*),13(*S*)-epoxy-linolenic acid (12(*S*),13(*S*)-HPOT) catalyzed by heme and then converted by propylene oxide cyclase (POC) to 12-oxo-phytodienoic acid (12-OPDA) [16]. 12-OPDA eventually produces JA after a series of reactions, which is a key step in the synthesis of JA in plants. Many studies have shown that the *AOS* in plants is closely related to the regulation of plant resistance to drought, salinity, low temperature, pests and diseases, strong UV radiation, and other adversities [17–20]. Many studies have shown that the expression of *AOS* genes are induced by various factors such as gibberellin (GA), abscisic acid (ABA), low temperature, salt, and drought stress, and they also play an important role in plant growth and development and in response to adversity stress [21–24]. There are two *AOS* homologs, *AOS1* and *AOS2*, in some plants, and the functions of *AOS1* and *AOS2* are the same, and they have different modes of action and different expression patterns [25].

In this study, we performed bioinformatics, expression analysis, and functional verification of *AOS* genes *HvnAOS1* and *HvnAOS2*. This study provides new sights for the function of *HvnAOS1* and *HvnAOS2* on the regulation of JA synthesis under integrated stress resistance in qingke.

## 2 Materials and methods

### 2.1 Amplification of *HvnAOS1* and *HvnAOS2* cDNA and promoter region

According to the searched domain of AOS in the plant genome database Gramene (http://www.gramene.org/), we identified *HvnAOS1* (HORVU4Hr1G066270) and *HvnAOS2* (HORVU4Hr1G066230) in qingke. Due to *HvnAOS1* and *HvnAOS2* without intron structure, the coding sequence of *HvnAOS1* (1,464 bp) and *HvnAOS2* (1,443 bp) and the sequence of their promoter regions were isolated from the qingke variety “Kunlun 15” genomic DNA using a KOD-FX high-fidelity PCR enzyme according to primers forward, 5′ AGTTAGATGAACCAGAG 3′ and reverse, 5′ GGATTTAAACAGCGTCTGCCACAC 3′; forward 5′ AGTGTAAGATGAACCAGAGCGC 3′ and reverse, 5′ AGGTTTTAAACAGCCCC 3′, respectively. The promoter region of the *HvnAOS1* (2,134 bp) and *HvnAOS2* (2,106 bp) were isolated by degenerate primers forward, 5′ TCAATGGTGTGCCATTATCTAC 3′ and reverse, 5′ CTAACTCTGCTGCTAGCTAGGCT 3′; forward, 5′ TAGTACCGCTCTGGCGGTACTGT 3′ and reverse, 5′ CTTACACTTGTTGCTTCAAAAT 3′, respectively. The DNA sequences of *HvnAOS1* and *HvnAOS2* genes and promoter region were cloned from leaf DNA as template by PCR. The open reading frames in the sequences were translated using DNAMAN (Version: 7.0) to obtain HvnAOS1 and HvnAOS2 protein sequences. The primer sequences used in this study were designed by Genscript (https://www.genscript.com.cn/tools/pcr-primers-designer).

### 2.2 Bioinformatics analysis

The promoter elements of *HvnAOSs* genes were predicted using the PlantCARE (http://bioinformatics.psb.ugent.be/webtools/plantcare/html/). The physical and chemical properties were predicted using the Protparam (https://web.expasy.org/protparam/). Secondary structures of HvnAOSs proteins were predicted using the SOPMA (https://npsa-prabi.ibcp.fr/cgi-bin/npsa_automat.pl?page=npsa_sopma.html). The alignments of nucleotide sequences and protein sequences were performed using DNAMAN 7.0 and the phylogenetic analysis was inferred with the maximum likelihood estimate method using the MEGA (Version: 7.0). Subcellular localizations of these HvnAOSs proteins were predicted using Cell-PLoc 2.0 (http://www.csbio.sjtu.edu.cn/bioinf/Cell-PLoc-2/). The protein structures were predicted using the AlphaFold (Version: 2.3.2). The comprehensive analysis of protein binding cavities was predicated by CavityPlus. The 13(*S*)-HPOT and Heme molecular PDB files were downloaded from the PDB database (https://www.rcsb.org/). Molecular docking simulations of HvnAOS1 and HvnAOS2 with 13(*S*)-HPOT and heme were performed with AutoDock Vina software (Version: 1.2) according to the study reported by Li et al. [16], respectively, and the conformation with the highest scoring value and default parameters were selected, and the results were analyzed using PyMoL software (Version: 1.7.6).

### 2.3 Subcellular localization analysis of the HvnAOS1 and HvnAOS2

The primers for vector construction are as follows: *HvnAOS1,* forward, F:5′-GCTCTAGAAGTTAGATGAACCAGAG-3′ and reverse, R:5′-CGGGTACCCAACAGCGTCTGCCACACC-3′; *HvnAOS2* F:5′-GCTCTAGAAGTGTAAGATGAACCAGAGCGC-3′ and reverse, R:5′-CGGGTACCCAACAGCCCCAGGACCGGATGTGGC-3 and the PCR fragment was cloned into the modified vector pBI221-GFP with Basta resistance gene at the Xba Ⅰ and KpnI sites. Then, pBI221:*HvnAOS1*-GFP, pBI221:*HvnAOS2*-GFP was transformed into leaf protoplasts of qingke for subcellular localization [26,27]. The qingke seeds were germinated at 25℃ in the dark for 10 days to cut the leaves into minced pieces (<0.5 mm). Protoplast preparation and transient transformation were carried out according to the instruction of Zhongke Terry Plant Protoplast Preparation and Transformation Kit (RTU4072). The green fluorescent protein (GFP) fluorescence signal of HvnAOS1 and HvnAOS2 was observed using the laser confocal scanning microscope (Nikon, C2-ER).

### 2.4 Expression pattern analysis of *HvnAOS1* and *HvnAOS2*

To analyze the expression levels of *HvnAOS1* and *HvnAOS2* under different phytohormone treatments, the qingke seeds were planted in soil-based seedling boxes and treated with hormones when they reached the three-leaf stage. A 100 µM solution of ABA, methyl jasmonate (MeJA), GA, salicylic acid (SA), 6-benzyl aminopurine (6-BA), and naphthalene acetic acid (NAA) was prepared and 5 mL of each solution was sprayed with 1‰ Tween-20, while the control plants were sprayed with only 200 mL of pure water with 1‰ Tween-20. The leaves were harvested and flash frozen in liquid nitrogen at 0 (control), 6, 12, 24, and 48 h after treatment and were stored at −80°C for RNA extraction. At least three biological replicates of each sample were performed to analyze the expression patterns of *HvnAOS1* and *HvnAOS2* [28,29]. Total RNA from the leaves was extracted using the TransGen Transzol Up Plus kit. The cDNA was synthesized using the first-strand cDNA synthesis super mix kit (Transgen Biotech, Catalog No. AE301-02). The pair of specific primers of *HvnAOS1* and *HvnAOS2* were as follows: *HvnAOS1*: forward, TTCGTCGGCGACCGGTTC and reverse, CTGCCACACCGGACG; *HvnAOS2*: forward, ACCGGTTTGTCGGGG and reverse, CCCCAGGACCGGAT. The primers of reference gene 18SrRNA were as follows: forward, CGGCTACCACATCCAAGGAA and reverse, GCTGGAATTACCGCGGCT. The qRT-PCR reaction system consisted of 1.0 μL primers (10 μM), 2.0 μL cDNA, 10 μL Thunderbird SYBR qPCR Mix, and 6.0 μL ddH_2_O. The formula 2^−ΔΔCt^ was used for qRT-PCR analysis and each reaction was repeated three times.

### 2.5 Transformation of *Arabidopsis* and identification of transgenic plants

To efficiently test whether the *HvnAOS1* and *HvnAOS2* are functionally similar to *Arabidopsis AOS*, we transformed *HvnAOS1* and *HvnAOS2* into the *Arabidopsis aos* mutant to test whether *HvnAOS1* and *HvnAOS2* can complement the JA-synthase-related phenotypes of *aos*, respectively. Recombinant vectors pBI221:*HvnAOS1*-GFP and pBI221:*HvnAOS2*-GFP were transformed into *Agrobacteria* strain GV3101 and then infected inflorescence of *Arabidopsis*, respectively. Since *Arabidopsis aos* is male-sterile [30,31], we sprayed *aos* with 100 µM MeJA every 3 days to restore fertility for *Agrobacterium* (GV3101)-mediated floral dip genetic transformation. Arabidopsis was cultured as described by An et al. [17]. Harvested Arabidopsis seeds were sterilized with bleach for 5 min and then washed 5–6 times with sterilized water. These seeds were selected on Basta (20 mg L^−1^) medium for the positive transgenic seedlings. The first generation of transgenic plants (T_1_) was confirmed by PCR amplification using *HvnAOS1* and *HvnAOS2*-specific primers, and T_2_ or T_3_ seeds were used for further research.

### 2.6 Identification of male fertility and *Botrytis cinerea* resistance in transgenic plant

Transgenic seedlings were grown on 1/2 MS medium containing 0.8% agar, 1% sucrose, and 20 mg L^−1^ Basta, wild-type (WT) seedlings were grown on same medium without Basta. After germination, the WT and transgenic *Arabidopsis* seedlings of similar size were selected and transferred to plant in 0.35-L pots with artificial mixed soil (pindstrup substrate: organic substrate: vermiculite = 5:4:1). The pots were placed under 12 h day and 12 h night cycles at 22°C for 25 days for the identification of male fertility and resistance*. Arabidopsis* and *B. cinerea* culture and spore suspension preparation were conducted as described by An et al. [17]. Three leaves of similar size were taken from WT, *aos*, *aos/HvnAOS1*, and *aos/HvnAOS2*. The leaves were inoculated with 10 μL of 10^7^ mL^−1^ spore suspension solution (0.025% Tween-20) and 15 μL control solution (autoclaved water and 0.025% Tween-20). The leaves were placed in a 90 mm diameter petri dish lined with moistened filter paper and sealed to keep the moisture in. Petri dishes were stored at 22°C. The diameter of the spots was counted after 2 days post-infection.

## 3 Results

### 3.1 Bioinformatics analysis of *HvnAOS1* and *HvnAOS2* genes

#### 3.1.1 Cis-acting elements analysis of the promoter region

The amplified promoter region sequences of *HvnAOS1* and *HvnAOS2* gene were analyzed by the PlantCARE software. A large number of TATA-box and CAAT-box promoter core elements as well as light-responsive cis-acting elements were found in the promoter regions. Only three ABA, one growth hormone, and one gibberellin responsive cis-acting elements were found in *HvnAOS1* promoter region (Table 1). In addition to cis-regulatory elements of anaerobic induction, fenestra cell differentiation, seed-specific regulation, and circadian rhythm control were also found in the promoter of *HvnAOS2* genes (Table 1).

**Table 1:** Analysis of cis-acting elements in *HvnAOS1* and *HvnAOS2* promoter region

Element	Motif	Function	Number	
			*HvnAOS1*	*HvnAOS2*
ABRE	ACGTG; GCCGCGTGGC; CGCACGTGTC	Cis-acting element involved in the ABA responsiveness	6	3
G-box	CACGTC; TACGTG	Cis-acting element involved in light responsiveness	11	11
AuxRR-core	GGTCCAT	Cis-acting regulatory element involved in auxin responsiveness	2	1
Box 4	ATTAAT	Cis-acting element conserved DNA module involved in light responsiveness	2	
CAAT-box	CAAAT; CCAAT	Common cis-acting element in promoter and enhancer regions	12	16
CAT-box	GCCACT	Cis-acting regulatory element related to meristem expression	1	2
CGTCA-motif	CGTCA; TGACG	Cis-acting regulatory element involved in the MeJA-responsiveness	6	
MRE	AACCTAA	MYB binding site involved in light responsiveness	1	
O2-site	GATGATGTGG	Cis-acting regulatory element involved in zein metabolism regulation	2	
TATA-box	TATA; TATAA; ATATAA; TATATA; TATATAAATC	Common cis-acting element in promoter and enhancer regions	29	23
TC-rich repeats	ATTCTCTAAC	Cis-acting element involved in defense and stress responsiveness	1	
TCA-element	CCATCTTTTT	Cis-acting element involved in SA responsiveness	1	
ARE	AAACCA	Cis-acting regulatory element essential for the anaerobic induction		1
P-box	TCTGTTG	Gibberellin-responsive element		1
HD-Zip 1	CAAT(A/T) ATTG	Element involved in differentiation of the palisade mesophyll cells		1
RY-element	CATGCATG	Cis-acting regulatory element involved in seed-specific regulation		1
Circadian	CAAAGATATC	Cis-acting regulatory element involved in circadian control		1

#### 3.1.2 Physicochemical properties and structure analysis of HvnAOS1 and HvnAOS2 protein

HvnAOS1 and HvnAOS2 proteins are composed of 487 and 480 amino acids, with molecular masses of 53,520.79 and 52,729.72 u, total atomic number of 7,602 and 7,464, hydrophilic coefficients of −0.015 for hydrophilic proteins and 0.028 for hydrophobic proteins, theoretical isoelectric points of 8.93 and 7.65, and instability indices of 33.75 and 32.20, respectively. The α-helix, extended strand, β-turn, and random coil of HvnAOS1 and HvnAOS2 proteins accounted for 39.43, 15.61, 5.43, 39.36, and 40.62% of the total amino acids, respectively, and 39.36, 40.62, 15.62, 4.38, 39.38%, respectively. Subcellular localization prediction revealed that HvnAOS1 and HvnAOS2 were localized in the endoplasmic reticulum (Table 2).

**Table 2:** Physicochemical properties and structural analysis of HvnAOS1 and HvnAOS2

Protein name	HvnAOS1	HvnAOS2
Molecular weight	53520.79	52729.72
Total number of atoms	7,602	7,464
Formula	C_2447_H_3821_N_631_O_691_S_12_	C_2410_H_3739_N_619_O_683_S_13_
GRAVY	−0.015	0.028
Theoretical pI	8.93	7.65
Instability index (II)	33.75	32.20
Aliphatic index	89.28	88.96
The proportions of α helix	39.43%	40.62%
The proportions of extended strand	15.61%	15.62%
The proportions of β turn	5.43%	4.38%
The proportions of random coil	39.36%	39.38%
Prediction of subcellular localization	Endoplasmic reticulum	Endoplasmic reticulum

#### 3.1.3 Phylogenetic tree construction

**Figure 1:**
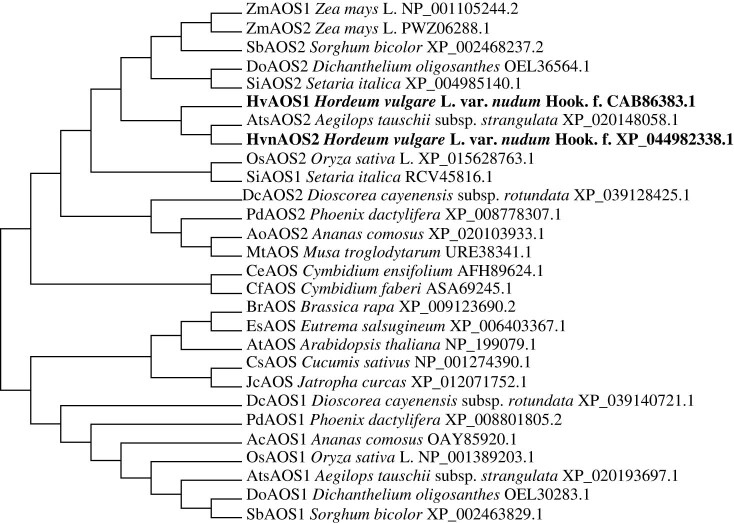
Phylogenetic tree of HvnAOS1 and HvnAOS2 with other species. Phylogenetic analysis of HvnAOS1 and HvnAOS2 with its homologs from other species. Phylogenetic analysis of AOS from dicotyledon and monocotyledon plants using MEGA7. Branches are labeled with GenBank accession numbers and the organisms.

The homologous protein sequences of HvnAOS1 and HvnAOS2 from qingke and 17 other plants were analyzed and their phylogenetic tree was constructed [32,33]. The results of phylogenetic tree showed that HvnAOS1 and HvnAOS2 had the highest similarity to AtsAOS2 from *Aegilops tauschii* subsp. *strangulata* (Figure 1).

#### 3.1.4 Amino acid sequence alignment and molecular docking simulation

**Figure 2:**
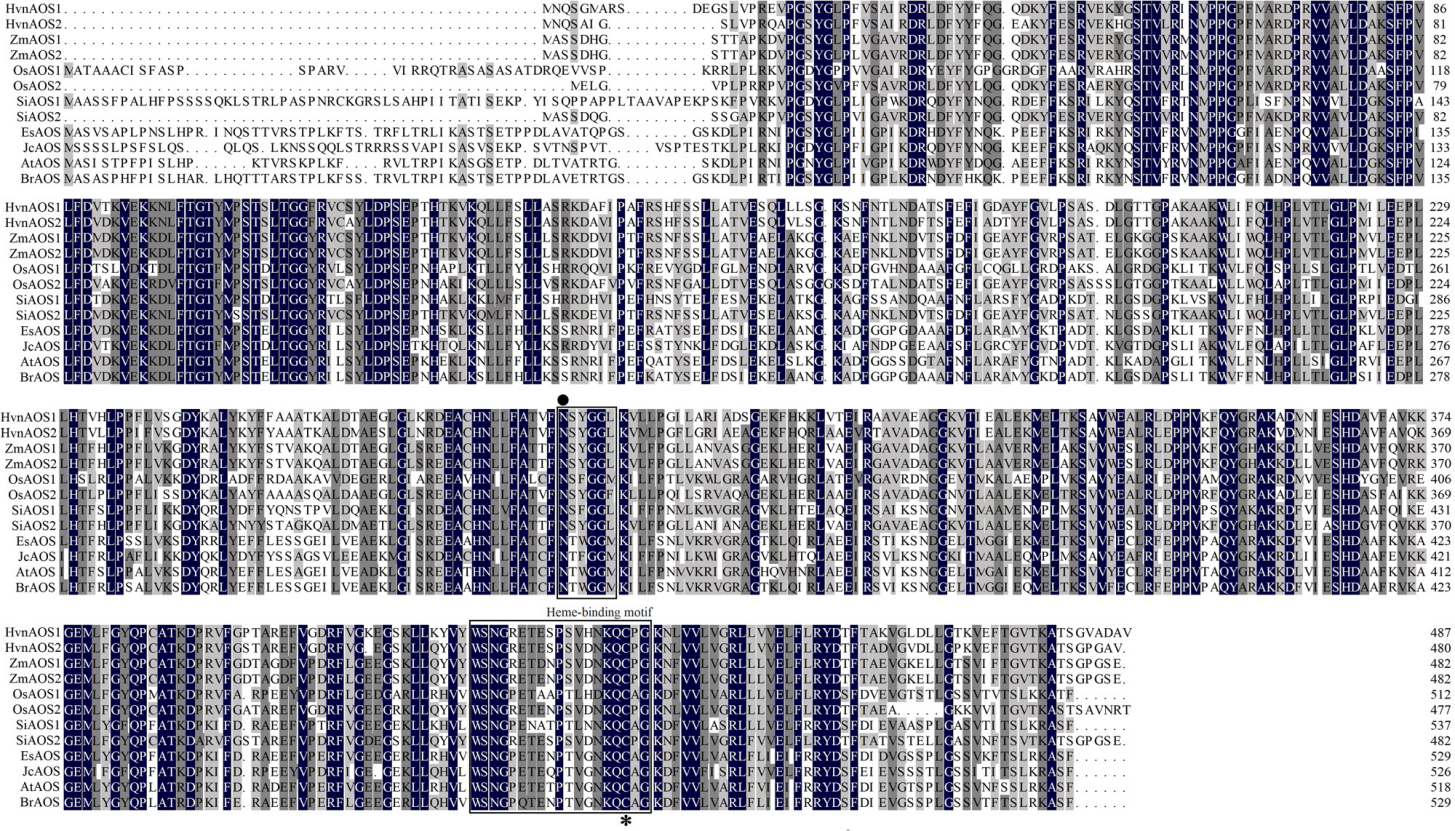
Protein sequence alignment of HvnAOS1 and HvnAOS2. *: The heme Fe^3+^ interactions key site for HvnAOS1 and HvnAOS2; ●: The key amino acid site for substrate catalytic.

**Figure 3:**
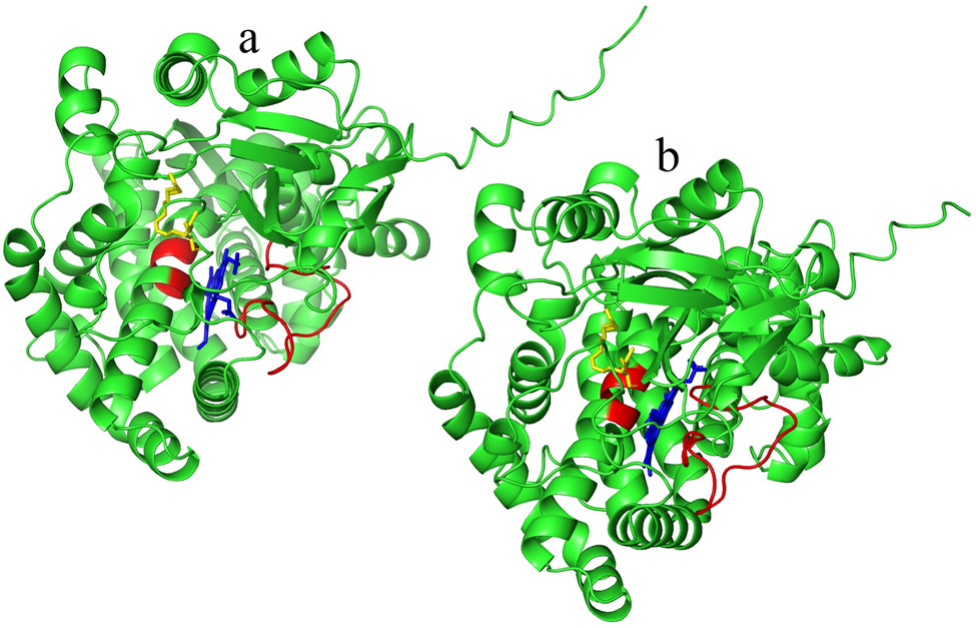
Molecular docking simulations of HvnAOS1 and HvnAOS2. (a) Model of HvnAOS1 (green molecule) docking with heme (blue molecule) and 13(*S*)-HPOT (yellow molecule). (b) Model of HvnAOS2 (green molecule) docking with heme (blue molecule) and 13(*S*)-HPOT (yellow molecule).

**Figure 4:**
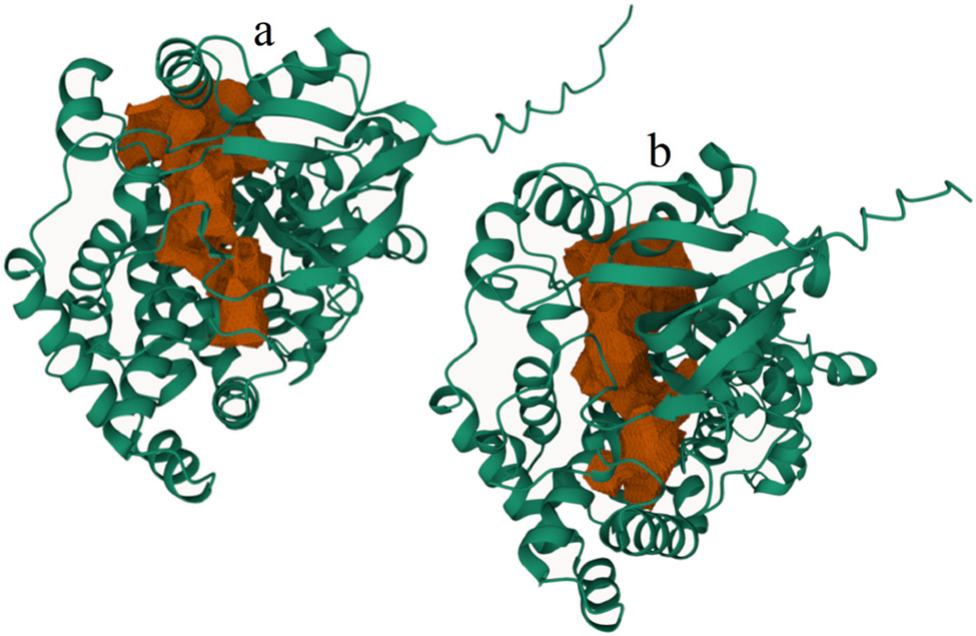
Protein binding cavities analysis of HvnAOS1 and HvnAOS2. (a) Protein binding cavities of HvnAOS1 (brown zone). (b) Protein binding cavities of HvnAOS2 (brown zone).

The protein sequence alignment of HvnAOS1, HvnAOS2, and other plant AOS homologs were performed using DNAMAN 7.0. The results showed that all AOS proteins have the conserved heme binding key region (Figures 2 and 3, red region), the substrate catalysis key site, and heme Fe^3+^ interactions key site (Figure 2). Protein binding cavity analyses revealed that the binding cavities of both HvnAOS1 and HvnAOS2 were located within the protein’s internal structure (Figure 4a and b [brown zone]).

**Figure 5:**
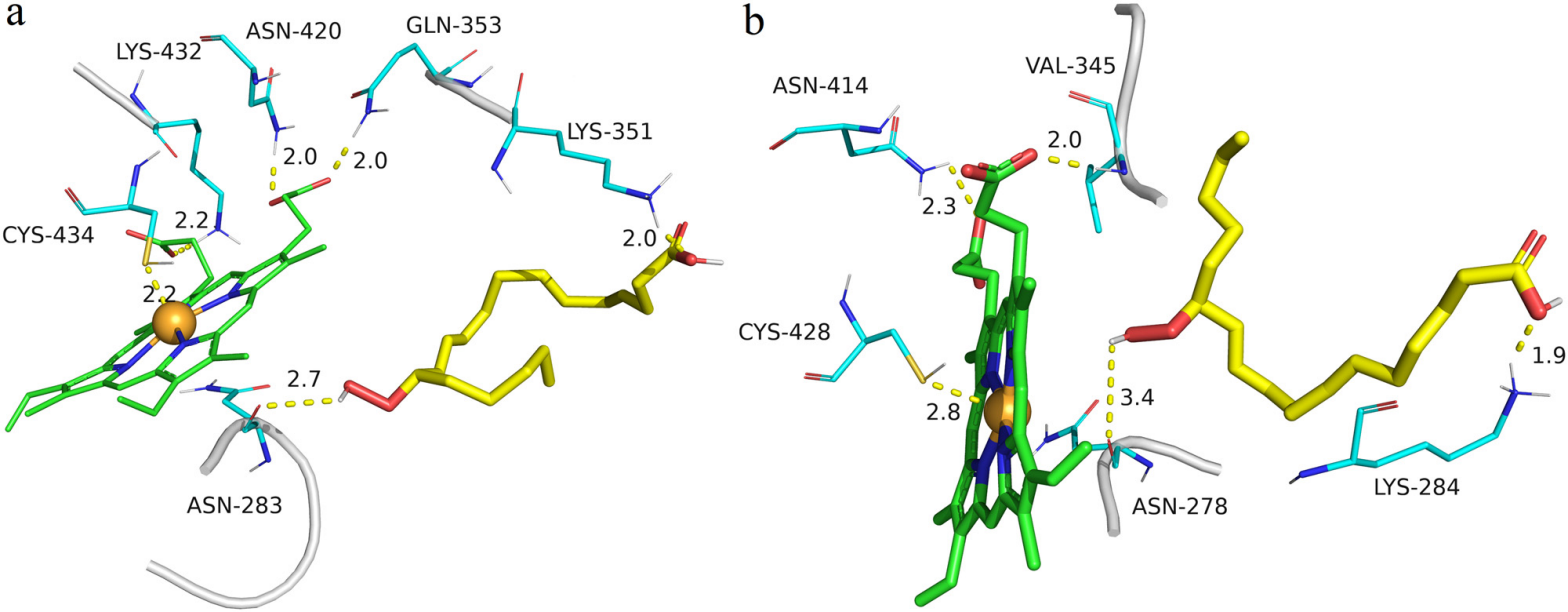
Key amino acid sites analysis of the HvnAOS1 and HvnAOS2 interact with heme and 13(*S*)-HPOT. (a) Key amino acid sites analysis of the HvnAOS1 interact with heme and 13(*S*)-HPOT. (b) Key amino acid sites analysis of the HvnAOS2 interact with heme and 13(*S*)-HPOT.

Molecular docking simulations revealed that one carboxyl group on heme in HvnAOS1 forms 2.0 Å hydrogen bonds with residues ASN420 and GLN353, respectively, and another carboxyl group on heme forms 2.2 Å hydrogen bonds with residue LYS432. The 13(*S*)-HPOT hydroxyl atom in HvnAOS1 forms 2.7 Å hydrogen bonds with residue ASN283 and 2.0 Å with residue and the Fe^3+^ in heme in HvnAOS1 forms a 2.2 Å covalent bond with the sulfhydryl sulfur atom of residue CYS434. However, a carboxyl group on heme in HvnAOS2 forms a 2.3 Å hydrogen bond with residue ASN414, and another carboxyl group on heme forms a 2.0 Å hydrogen bond with residue VAL345. The 13(*S*)-HPOT hydrogen atom in HvnAOS2 (*S*)-HPOT hydroxyl hydrogen atom forms a 3.4 Å hydrogen bond with residue ASN278 and a 1.9 Å hydrogen bond with residue LYS284. The Fe^3+^ in heme forms a 2.8 Å covalent bond with the sulfhydryl sulfur atom of residue CYS428. These interactions are essential for HvnAOS1 and HvnAOS2 to bind the substrate 13(*S*)-HPOT and the catalytic factor heme (Figures 3 and 5).

### 3.2 Subcellular localization of HvnAOS1 and HvnAOS2

**Figure 6:**
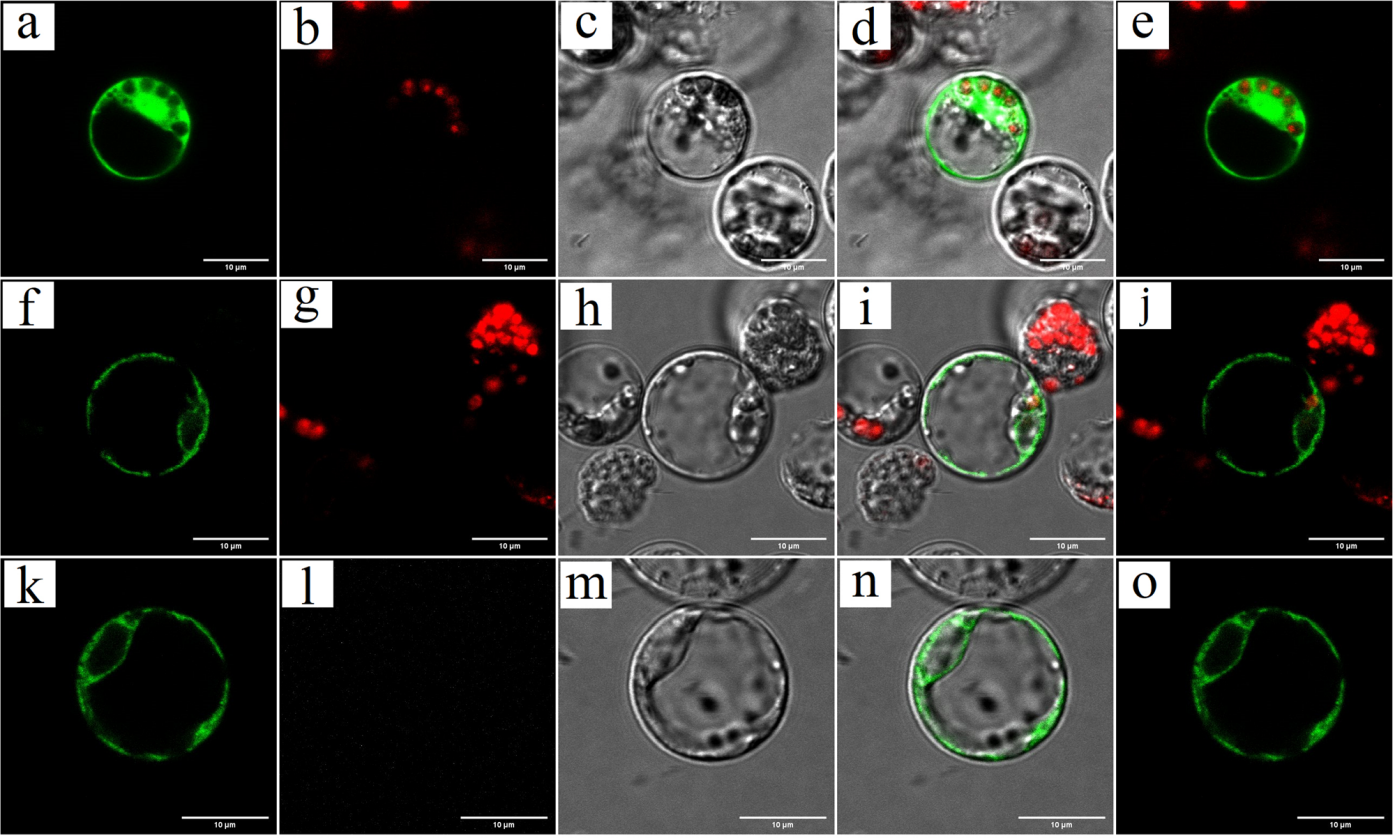
Subcellular localization of HvnAOS1 and HvnAOS2 in leaf protoplasts of qingke. HvnAOS1-GFP and HvnAOS2-GFP or GFP alone were transiently expressed in leaf protoplasts of qingke and GFP signals were examined by Laser Scanning Confocal Microscopy (Nikon C2-ER). GFP (a)–(e) the GFP fluorescence, chlorophyll autofluorescence, bright field, bright field merged, and dark field merged of 35S: GFP. (f)–(j) GFP fluorescence, chlorophyll autofluorescence, bright field, bright field merged, and dark field merged of 35S: HvnAOS1-GFP. (k)–(o). GFP fluorescence, chlorophyll autofluorescence, bright field, bright field merged, and dark field merged of 35: HvnAOS2-GFP.

To understand the localization of HvnAOS1 and HvnAOS2 in plant, HvnAOS1:GFP and HvnAOS2:GFP were transiently expressed in protoplast. Subcellular localization of HvnAOS1 and HvnAOS2 revealed that the GFP signal was observed in cytoplasm and fibrillary. In summary, these results suggested that HvnAOS1 and HvnAOS2 were accumulated in the endoplasmic reticulum in combination with the results of prediction (Figure 6).

### 3.3 Expression pattern analysis of *HvnAOS1* and *HvnAOS2* by plant hormone treatment

**Figure 7:**
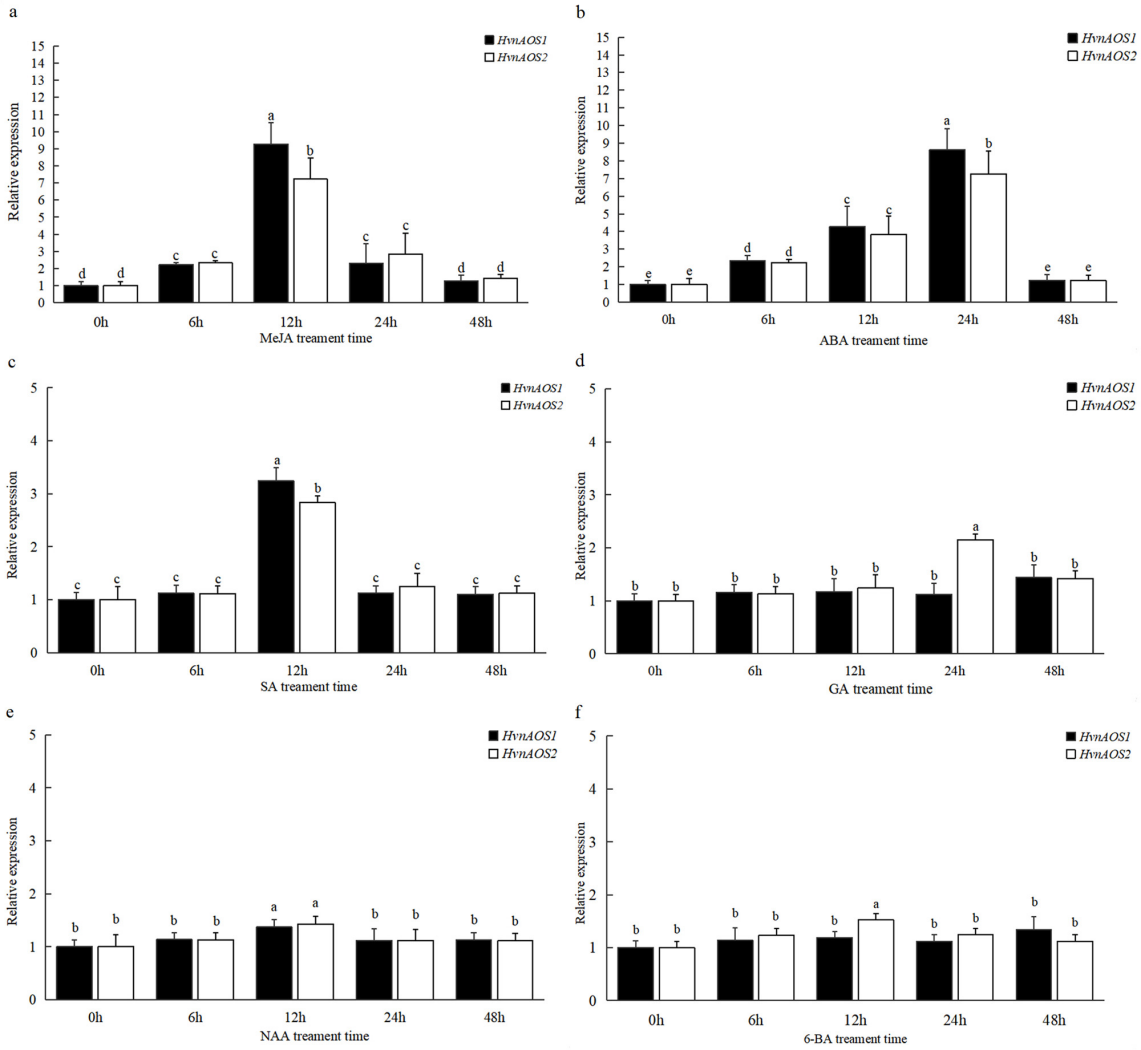
Expression profile of *HvnAOS1* and *HvnAOS2* in response to exogenous hormones: (a)–(f) MeJA, methyl jasmonate; ABA, abscisic acid; SA, salicylic acid; GA, gibberellin; NAA, naphthalene acetic acid; 6-BA, 6-benzyl aminopurine. Real-time PCR data were analyzed using the comparative 2^−ΔΔCt^ method to quantify relative gene expression.

In view of many cis-acting elements related to plant hormones in *HvnAOS1* and *HvnAOS2* promoter region, we tested whether *HvnAOS1* and *HvnAOS2* were induced by plant hormones in the leaves. The results showed that *HvnAOS1* and *HvnAOS2* genes were strongly induced in the leaves with JA, ABA, and SA treatments, but there was no significant induction in the leaves with GA, NAA, and 6-BA treatments (Figure 7).

### 3.4 Over-expression of *HvnAOS1* and *HvnAOS2* in *Arabidopsis aos* mutant restores male fertility and causes immunity recovery against *B. cinerea*

**Figure 8:**
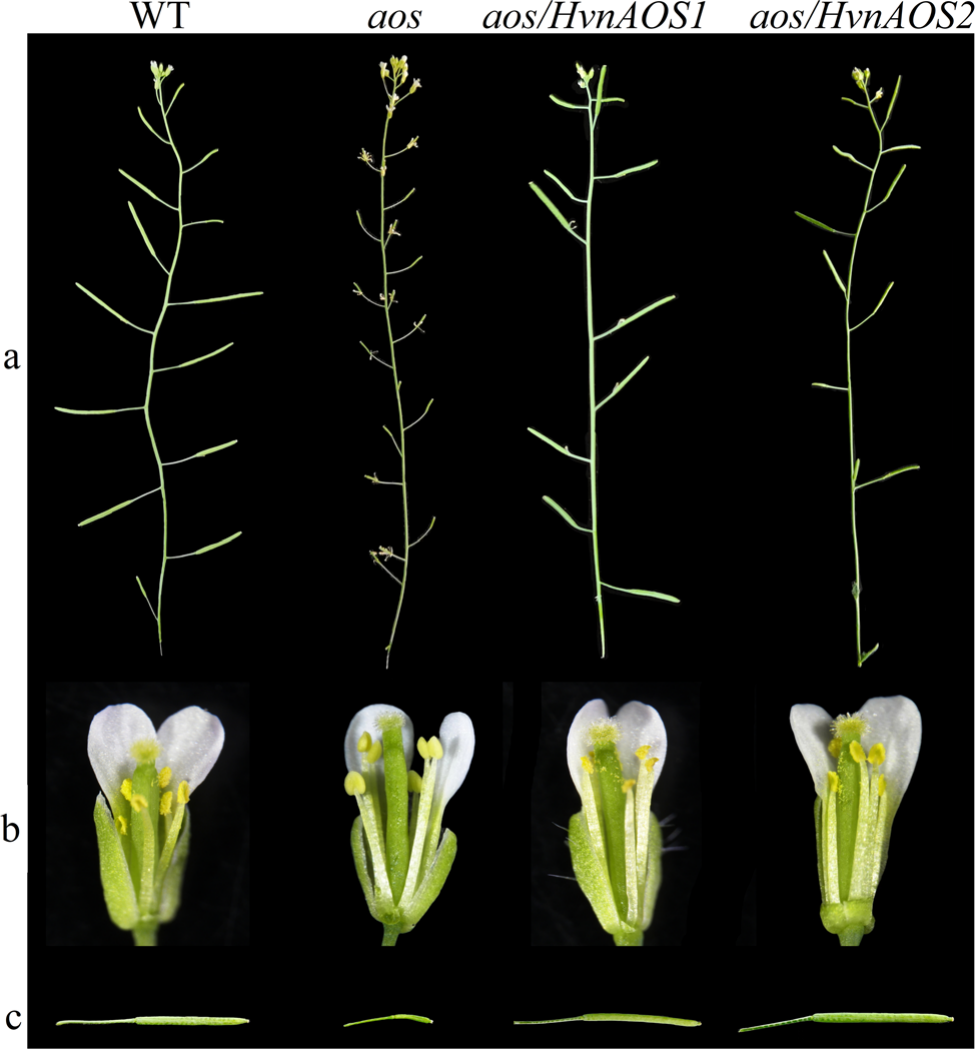
Flower morphological phenotypes of *aos* mutants transformed with *HvnAOS1* and *HvnAOS2*, respectively, at the T_1_ generation. (a) Inflorescences of WT, *aos*, and transformants. (b) Flowers of WT, *aos*, and transformants. (c) Fully developed siliques of WT, *aos,* and transformants.

To efficiently test whether *HvnAOS1* and *HvnAOS2* are functional as AOS, we transformed *HvnAOS1* and *HvnAOS2* gene into the *Arabidopsis aos* mutant, respectively, and tested whether *HvnAOS1* and *HvnAOS2* can complement the JA-insensitivity-related phenotypes of *aos*. As described earlier, *Arabidopsis* aos mutant exhibited male sterility (Figure 8). However, over-expression of HvnAOS1 and HvnAOS2 in *Arabidopsis aos* mutant restored its male fertility, indicating that HvnAOS1 and HvnAOS2 are capable of the function on male fertility.

**Figure 9:**
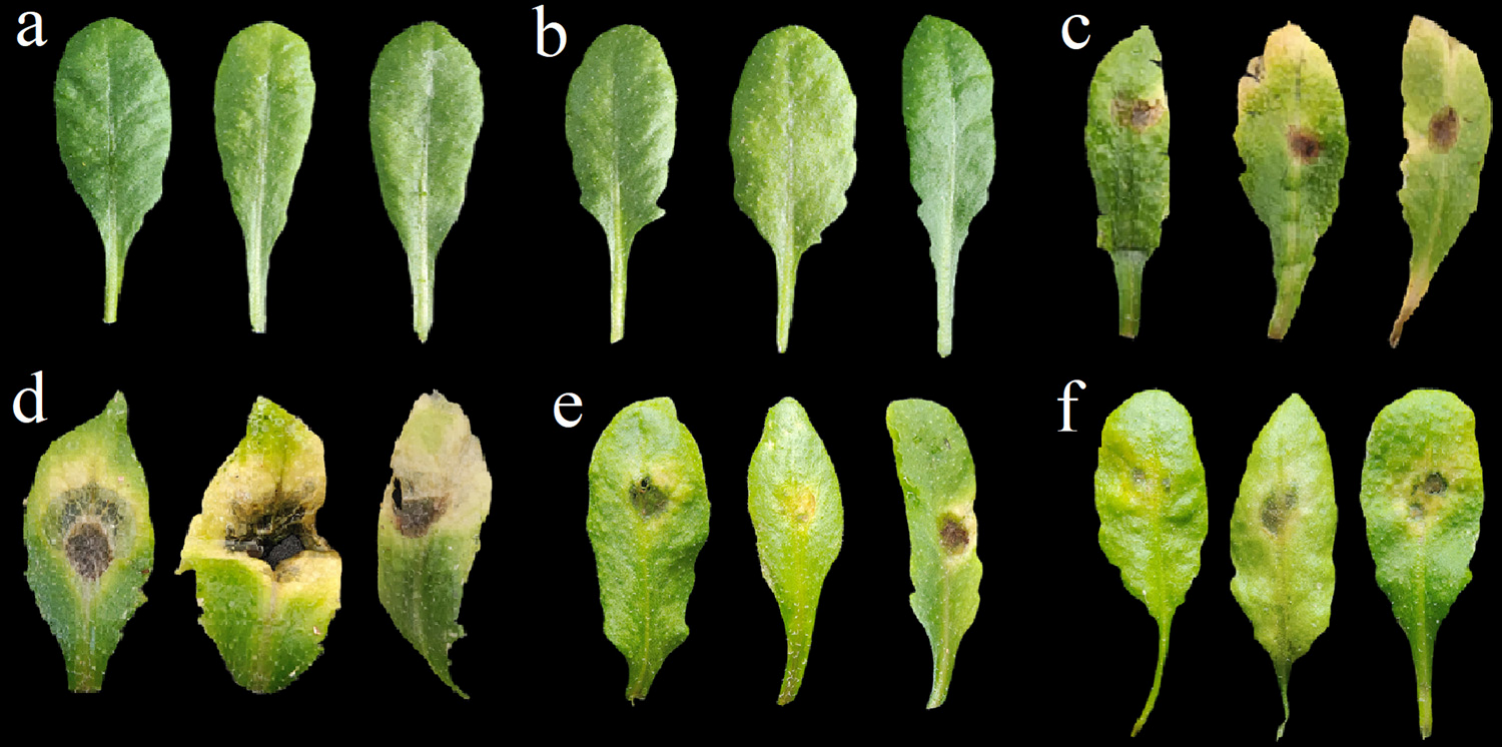
Susceptibility of *aos* transformants with *HvnAOS1* and *HvnAOS2*, respectively, to *B. cenerea*: (a) WT control, (b) *aos* control, (c) WT inoculated with *B. cenerea*, (d) *aos* inoculated with *B. cenerea*, (e) *aos/HvnAOS1*, and (f) *aos/HvnAOS2* inoculated with *B. cenerea*.

JA is one of the major defense hormones in plants, and the *Arabidopsis* JA biosynthesis mutant *aos* are highly susceptible to the necrotrophic pathogen *B. cinerea*. In this study, we tested whether *HvnAOS1* and *HvnAOS2* are able to complement the function on defense against *B. cinerea* in the *aos* mutant. When inoculated *B. cinerea* on *Arabidopsis* leaves, the *aos* mutant exhibited high susceptibility to *B. cinerea* (Figure 9). However, the *HvnAOS1* and *HvnAOS2* complementation on the *aos* mutant were similar to the wild type *Arabidopsis* in terms of resistance (Figure 9). Taken together, *HvnAOS1* and *HvnAOS2* share the similar function with *Arabidopsis AOS* gene on male fertility and plant resistance to *B. cinerea.*

## 4 Discussion

AOS is one of the crucial synthase enzymes in the JA biosynthesis pathway in plants, belonging to the CYP74A family within the cytochrome P450 supergene family [34–37]. Due to its involvement in the JA-related stress responses, growth developmental regulation, and hormone signaling pathways in plants, *AOS* genes have been a prominent subject of research in different plant species. Additionally, *AOS* plays distinct physiological roles in plants, further highlighting its significance. As a result, *AOS* remains a focal point of investigation, with numerous unknown functions awaiting discovery [38]. There are currently very few studies on the structure, expression pattern, and function of *AOS*, and only a few studies have been reported in Arabidopsis and rice, so it is particularly important to study the structure, expression pattern, and function of *HvnAOS1* and *HvnAOS2*.

In this study, we cloned *HvnAOS1* and *HvnAOS2* genes and their promoter regions, and analyzed their bioinformatics, expression patterns, and functions. The elements in the promoter regions of *HvnAOS1* and *HvnAOS2* are significantly different. They are likely to have different expression patterns and functions under different conditions. The *HvnAOS1* promoter region contains a number of phytohormone-responsive cis-elements, whereas the promoter region of *HvnAOS2* lacks phytohormone-responsive elements. Instead, it contains specific cis-regulatory elements related to anaerobic induction, mesophyll cell differentiation, seed-specific regulation, and diurnal rhythm control. *HvnAOS1* is likely to be involved in JA synthesis under normal barley conditions or in common stress responses. On the other hand, *HvnAOS2* may play a role in JA synthesis in specific tissues or under specific conditions. *HvnAOS1* and *HvnAOS2* have similar physicochemical properties, but HvnAOS1 is hydrophilic whereas HvnAOS2 is hydrophobic.

The predication results indicated that HvnAOS1 and HvnAOS2 were found to be located in the endoplasmic reticulum. However, some studies have found that the subcellular localization of AOS were different among different plants. Most plant AOS possess typical chloroplast transit peptides and are primarily located in chloroplasts. However, recent studies indicate that barley AOSs does not have chloroplast transit peptides, and AOSs in some plants can be detected within specific organelles such as PhAOS from *Parthenium hysterophorus* L. which is localized in intracellular rubber particles [39,40].

Molecular docking simulation of the key residues in the different interaction patterns of HvnAOS1 and HvnAOS2 indicated that HvnAOS1 interacts with 13(*S*)-HPOT at residue ASN283 and with heme Fe^3+^ at residue CYS434. HvnAOS2 interacts with 13(*S*)-HPOT at residue ASN278 and with heme Fe^3+^ at residue CYS428. Interestingly, these two key interaction sites are the same amino acid residues in both HvnAOS1 and HvnAOS2. Li et al. [16] investigated the crystal structure of PaAOS and iron porphyrin from *Parthenium argentatum* and its complex with the substrate analogue 13(*S*)-HODE and its intermolecular interactions, and found that the ASN-276 residue in PaAOS is in the active site and very close to heme, and that this amino acid site is highly conserved among AOS from different species, ASN-276 is thought to play an important role in substrate catalysis, and the CYS-426 residue forms a covalent bond with Fe^3+^ in the center of heme, which is thought to play an important role in the binding to heme. The results in this study are consistent with the reports, which suggest that these interactions are highly conserved in AOS and are important sites of action for AOS function. In addition, *HvnAOS1* and *HvnAOS2* are capable of complementing the Arabidopsis *aos* mutant, suggesting that *HvnAOS1* and *HvnAOS2* have similar functions as the Arabidopsis *AOS* gene.

In conclusion, we have identified two *AOS* genes *HvnAOS1* and *HvnAOS2* from hulless barley “Kunlun 15” that controlled the dehydration of fatty acid hydroperoxides to allene oxides. *HvnAOS1* and *HvnAOS2* were highly induced by JA, ABA, and SA. HvnAOS1 and HvnAOS2 mainly accumulated in the cell cytoplasm of the *N*. *benthamiana* leaves. Overexpression of *HvnAOS1* and *HvnAOS2* in Arabidopsis *aos* mutant restored male fertility and plant resistance to *B. cinerea*, indicating that *HvnAOS1* and *HvnAOS2* can restore the functions of *AOS* in Arabidopsis *aos* mutant. This study will provide a reference for research on the acid oxydase synthase and regulation of JA synthesis in qingke. It is important to note that the specific *AOS* genes and their functions can vary among different plant species, and further research is needed to explore the diversity and functional significance of *AOS* genes in various plants.
